# What are the odds? Identifying factors related to competitive success in powerlifting

**DOI:** 10.1186/s13102-022-00505-2

**Published:** 2022-06-19

**Authors:** Daniel J. van den Hoek, Patrick J. Owen, Joel M. Garrett, Robert J. Howells, Joshua Pearson, Jemima G. Spathis, Christopher Latella

**Affiliations:** 1 School of Behavioural and Health Sciences, Faculty of Health Science, Australian Catholic University, 1100 Nudgee Rd, Banyo, Brisbane, QLD 4014 Australia; 2 Institute for Physical Activity and Nutrition (IPAN), School of Exercise and Nutrition Sciences, Deakin University, 75 Pigdons Rd, Geelong, VIC Australia; 3 School of Medical and Health Sciences, Centre for Human Performance, Edith Cowan University, 270 Joondalup Dr, Joondalup, WA Australia; 4 Neurophysiology Research Laboratory, Edith Cowan University, 270 Joondalup Dr, Joondalup, WA Australia

**Keywords:** Athletic performance, Bench press, Competitive success, Deadlift, Strength sport, Squat

## Abstract

**Background:**

The ability for athletes to gain a competitive advantage over their opponents is well recognised. At times, this advantage may be considered a marginal gain. However, in the context of competition, marginal advantages may be the difference between winning and losing. This investigation explores how competition factors influence the odds of competitive success (i.e. winning) in powerlifting (PL) to assist athletes and coaches in achieving a competitive advantage.

**Methods:**

A cross-sectional, retrospective analysis of competition data from raw/classic, Australian powerlifting competitions 2010–2019 was conducted. Data included 10,599 competition entries (males: n = 6567 [62%], females: n = 4032 [38%]). Independent t-tests were used to compare continuous data between sexes or winners and non-winners at an event. Cohen’s d and the 95% confidence interval (d [95% CI]) were calculated. Univariate odds of winning an event based on independent variables (age [irrespective of category], sex, body weight and weight of first lift attempt [regardless of success]), were assessed by separate simple logistic regression.

**Results:**

When compared to males, the odds of winning for females were 50% greater (OR [95% CI] 1.500 [1.384, 1.625]; P < 0.001). Athletes who had larger first lift attempts (Squat: + 7.0 kg P < 0.001, Bench Press: + 3.2 kg P < 0.001, and Deadlift: + 6.1 kg P < 0.001and competed for a longer period (winners: 401 vs non-winners: 304 days, P < 0.001) had an increased likelihood winning. Age was associated with increased odds of success for males (OR [95% CI] 1.014 [1.009, 1.019], P < 0.001) per additional year of age for males, but not females (P = 0.509).

**Conclusions:**

Multiple factors appear to contribute to the likelihood of winning a PL competition. These results may help coaches to develop competition and training strategies that optimise athletes’ likelihood of competitive success in PL.

## Introduction

Powerlifting (PL) is a strength sport in which athletes generally aim to lift the heaviest weight they are capable of in the squat (SQ), bench press (BP) and deadlift (DL). During competition, athletes compete in an order, determined by the weight chosen for each lift attempt from lightest to heaviest [1]. Each athlete is given three attempts at each lift and must achieve at a minimum, one successful lift in each of the three lift-types (e.g. SQ, BP and DL), otherwise, the athlete does not achieve a ‘total’ and is disqualified from the competition. Comparable with many other strength-based sports, PL competitions are categorized by sex, weight categories and age groups.

Previous research has examined the attributes of successful PL athletes in relation to their training practices [2–4], physiological and anthropometric characteristics [5–7] and recently, the temporality of competition [8]. Whilst it is apparent that strength gains and the expression of maximal strength can be manipulated through favourable physiologic and/or training variables [6, 8, 9], limited studies explore strength expression and factors predicting success during competition [10–12]. Understanding these variables is important, as with many sports, tactical decisions, and strategy in the lead up to, and during competition can assist in developing a competitive advantage that is not always measurable via more traditional anatomical and physiological assessment protocols. Thus, emerging research within the PL community has begun to analyse competition data to predict, strategize and enhance the performance of athletes on the day of the contest [8–11, 13, 14], but further investigation is required to appropriately report and inform competitive practice of PL athletes and coaches.

Typically, elite PL athletes achieve a higher number of successful attempts for each of the three lifts than their novice counterparts [10]. For example, during the 2012 Oceania and 2013 Classic World Championships the successful completion of the first attempt for each of the SQ, BP and DL was greater for those who placed in the top three of their respective weight class and discipline (i.e. SQ, BP or DL) compared to those who did not place [15]. Moreover, 57% of medal finishers at the world-level successfully completed eight out of nine lifts during competition [15]. However, another study by Coker et al. [10] reported that a successful first attempt for the SQ, BP or DL did not result in greater odds of achieving a successful third attempt for these lifts, or a successful SQ or DL in the subsequent attempt. The relationship between successful opening attempts and overall competition outcome have not been well explored. Limited information has also been presented regarding the weight of each attempt, and evidence regarding subsequent competition outcomes is scarce. For example, although Travis et al. [11] explored the opening attempt weight selections of elite raw powerlifters in the International Powerlifting Federation (IPF), this investigation included only athletes who completed nine successful lift attempts but did not distinguish finishing positions within the competition. Collectively, the current literature does not appear to identify winning weight attempts for each of the three lifts, nor does it attempt to describe the competition day factors which correlate with competitive success.

The aims of this retrospective cross-sectional investigation were to 1. determine differences in weights achieved by winning and non-winning raw powerlifting athletes, and 2. explore how age, sex, body weight, time competing and relative opening attempt weight (SQ, BP and DL) influence the likelihood of successful competition performance (i.e. winning). We hypothesised that opening attempts of winners (relative and absolute weight) for each lift would be greater than those who lost. Additionally, we hypothesised that winners would have competed for a significantly longer period, and that age (across the total sample), and body weight (within classes) would not significantly differ between those who won and those who lost. Identification and exploration of modifiable competition factors (i.e., attempt weight selection, and increase in weight between attempts) may help coaches to develop better strategies and make informed tactical decisions related to the athlete or competition in an attempt to increase the odds of competitive success. The effects of non-modifiable factors on competitive success may also guide coaches in prioritising their selection and programming of athletes seeking competitive success.

## Methods

Powerlifting competition records were collated from November 28th, 2010–August 11th, 2019. Data were extracted from publicly available databases; Powerlifting Australia (www.powerliftingaustralia.com/results) and Open Powerlifting (www.openpowerlifting.org). Given the retrospective design and public nature of the competition results, an ethics waiver was granted by the Human Research Ethics Committee. All methods were carried out in accordance with relevant guidelines and regulations.

### Participants

Data were collated from male and female competitors registered and competing in raw competitions with Powerlifting Australia during 2010–2019. All individuals/parents/guardians provided informed consent to the use of competition data at the time of registration of membership with Powerlifting Australia. Within the available dataset of 11,816 competition entries, age was unavailable for 1198 competitors and first lift weight from the SQ, BP or DL was unavailable for 89 competitors and thus, these data were omitted from analysis. Consequently, 10,599 competition entries (males: n = 6567 [62%], females: n = 4032 [38%]) across 353 competition meets were included in all analyses.

### Procedures

To be included within the analysis, athletes must have competed at least once from 2010 to 2019 at a ‘classic’ Powerlifting Australia sanctioned event. Notably, Powerlifting Australia changed affiliation from the International Powerlifting Federation (IPF) to World Powerlifting (WP) in late 2017. As each organisation has slightly different weight classes, these have all been reported independently. Lift attempts for each of the three competitive lifts along with ‘total’ competition scores and category (i.e. age, weight class and sex) and competition result (i.e. competitive placing) for each competitor were extracted from official competition results. ‘Total’ score is the cumulative score of the best successful lift in kilograms from all three disciplines: SQ, BP and DL at each competition. In addition, database errors were removed by manually screening and determination of outliers. Winners were identified as those who placed first in their given age and weight class at a given meet. Non-winners were those who finished in second place or lower at these meets. These data do not include results from single lift or equipped events. Each competition had one entrant who won, yet entrants per competition (and number of those who did not win) varied (maximum: 21 entrants, mean [SD]: 9 [7] entrants, median [inter-quartile range]: 12 [14] entrants). Some athletes (i.e. those who competed more than once within the data capture period) are included multiple times within the dataset. The authors acknowledge that this was a violation of the independence assumption within these analyses. We report the percentage of competitors achieving their maximum successful attempt at attempt three for each of the SQ, BP and DL. Additionally, athlete age at time of competition was calculated from reported date of birth and competition date within the data set. Length of time competing for athletes was calculated as the time (in days) from each individual athlete’s first competition to their last within the dataset. Weight of the first lift attempt was considered as both absolute (kg) and relative to entrant body weight (weight of lift in kg divided by body weight).

### Statistical analysis

All analyses were conducted using Stata statistical software version 16 (College Station TX, USA). Independent t tests were used to compare total sample continuous data (age, length of time competing, number of competitions participated, body weight, weight of first lift attempt [regardless of success]) between sexes (male/female) or winning an event (yes/no). Cohen’s *d* and the lower and upper 95% confidence interval (*d* [95% CI]) were calculated and interpreted as: *d* ≤ 0.2 = small effect; *d* 0.2 ≥ 0.5 = medium effect; *d* ≥ 0.8 = large effect [16]. Univariate odds of winning an event (i.e. OR) based on independent variables (age [irrespective of category], sex, body weight and weight of first lift attempt [regardless of success]) were assessed by separate simple logistic regression and expressed as a ratio or percentage. An alpha level of 0.05 was adopted for all statistical tests. No adjustment was made for multiple comparisons [17].

## Results

Athlete demographics for all competition entries are shown in Table 1. In the total sample, when compared to males, females were 3 years older (P < 0.001, *d* = 0.31 [0.27, 0.35]) and had 25% (20.0 kg) lower body weight (P < 0.001, *d* = 1.15 [1.11, − 1.19]). When compared to those who lost, those who won were 1 year older (P < 0.001, *d* = 0.12 [0.08, 0.16]). Those who won had also been competing for a longer duration of time than those who lost: total sample (401 vs. 304 days, P < 0.001, *d* = 0.19 [0.15, 0.23]), females (369 vs. 278 days, P < 0.001, *d* = 0.19 [0.12, 0.25]), males (427 vs. 318 days, P < 0.001, *d* = 0.21 [0.16, 0.26]). Similarly, those who won had participated in more competitions. This was true for the total sample (3.40 vs. 2.75 competitions, P < 0.001, *d* = 0.23 [0.19, 0.27]), males (3.41 vs. 2.79 competitions, P < 0.001, *d* = 0.23 [0.18, 0.28]) and females (3.38 vs. 2.67 competition, P < 0.001, *d* = 0.25 [0.19, 0.31]).

**Table 1:** Demographics of all male and female competition entries who won and lost. Data are mean (standard deviation)

Variable	Male (n = 6,567)	Female (n = 4,032)	Win (n = 4,199)	Lose (n = 6,400)
Age, year	**28 (10)**^**‡**^	31 (11)	**30 (11)**^**‡**^	29 (11)
Body weight, kg	**89.5 (18.3)**^**‡**^	69.5 (15.7)	81.7 (22.6)	82.0 (17.8)
Age ≥ 40 year^#^	**725[11%]**^**‡**^	784 [19%]	642 [15%]	867 [14%]
Time competing, days	**357 (511)**^**‡**^	318 (489)	**401 (528)**^**‡**^	304 (483)
Average competitions, n	3.0 (2.9)	3.0 (2.8)	**3.4 (3.3)**^**‡**^	2.8 (2.5)

^*^P < 0.05; ^†^*P* < 0.01; ^‡^P < 0.001

The average, maximum, successful lift for the SQ, BP and DL in each weight class (IPF and WP classifications) is displayed in Table 2. For females, the average, maximum, successful attempt weight for the SQ (*d* = 0.46 [0.39, 0.52]), BP (*d* = 0.45 [0.39, 0.51]) and DL (*d* = 0.48 [0.41, 0.54]) were greater than for those who lost (P < 0.001). The same was true for males (SQ *d* = 0.43 [0.38, 0.48]; BP *d* = 0.43 [0.38, 0.48]; DL *d* = 0.46 [0.41, 0.51] P < 0.001), however, this trend was not seen for all weight classes (see Table 2).

**Table 2:** Average absolute (kg) maximum successful lift of those who won and lost in IPF and WP sanctioned competitions

Weight class	Squat		Bench press		Deadlift	
	Won	Lost	Won	Lost	Won	Lost
Female	**117.45 (27.28)**^**‡**^	106.01 (22.81)	**65.31 (14.72)**^**‡**^	59.18 (12.70)	**141.88 (25.98)**^**‡**^	130.20 (23.11)
47 kg^^^	87.22 (19.02)	78.10 (16.24)	51.02 (11.61)	48.93 (13.15)	113.32 (16.28)*	102.13 (20.44)
48 kg^#^	98.78 (21.87)	98.38 (12.32)	58.72 (11.81)	56.75 (10.91)	125.31 (23.36)	122.38 (15.28)
52 kg^^^	**100.23 (20.44)**^**‡**^	91.68 (16.95)	**56.62 (11.81)**^**†**^	52.07 (9.67)	**123.94 (21.70)**^**†**^	115.40 (19.76)
53 kg^#^	99.51 (16.47)	95.75 (17.46)	55.01 (10.73)	52.15 (9.99)	124.14 (18.95)	123.52 (21.64)
57 kg^^^	105.51 (20.86)	96.12 (20.51)	**61.52 (12.53)**^**‡**^	55.16 (11.85)	**132.31 (21.62)**^**‡**^	122.00 (23.32)
58 kg^#^	**108.12 (18.91)**^**†**^	99.19 (13.76)	61.08 (13.35)*	56.44 (10.04)	**136.93 (20.44)**^**†**^	126.37 (17.36)
63 kg^^^	**113.10 (22.61)**^**‡**^	101.69 (19.53)	**63.84 (13.41)**^**‡**^	56.84 (11.20)	**140.09 (24.31)**^**‡**^	126.54 (20.58)
64 kg^#^	**120.84 (25.37)**^**‡**^	108.00 (21.68)	**65.96 (13.64)**^**‡**^	58.59 (11.59)	**146.97 (27.03)**^**‡**^	130.65 (24.04)
72 kg^#^^	**122.91 (23.12)**^**‡**^	108.27 (21.58)	**66.70 (12.69)**^**‡**^	59.40 (11.89)	**148.37 (24.34)**^**‡**^	132.05 (22.29)
84 kg^#^^	**126.28 (24.49)**^**‡**^	111.20 (25.03)	**69.68 (14.89)**^**‡**^	62.59 (14.06)	**149.49 (23.50)**^**‡**^	135.30 (23.53)
84 kg+^^^	**133.46 (32.96)**^**‡**^	117.64 (25.54)	**72.49 (15.92)**^**‡**^	66.96 (13.88)	**154.35 (27.75)**^**‡**^	140.81 (22.89)
100 kg^#^	133.79 (29.12)*	119.44 (27.00)	**75.39 (16.48)**^**†**^	64.54 (13.70)	153.00 (24.08)	142.69 (27.52)
100 kg+^#^	138.77 (37.43)	127.60 (17.89)	74.55 (15.15)	71.53 (5.19)	145.00 (20.74)	139.63 (12.81)
Male	**202.47 (48.84)**^**‡**^	184.34 (37.92)	**133.04 (31.73)**^**‡**^	120.89 (25.91)	**235.15 (44.52)**^**‡**^	217.08 (36.44)
59 kg^^^	137.81 (34.88)	144.37 (44.43)	91.28 (19.08)	88.56 (15.73)	170.49 (32.04)	167.62 (35.31)
62 kg^#^	114.80 (38.63)*	181.00 (83.44)	79.85 (28.03)	100.13 (25.30)	156.60 (42.00)	179.00 (35.68)
66 kg^^^	155.79 (32.04)	151.14 (27.73)	100.30 (20.22)	96.81 (20.16)	191.15 (34.19)*	183.04 (28.11)
69 kg^#^	155.70 (36.21)	157.28 (32.27)	98.47 (18.34)	98.88 (20.43)	191.00 (38.85)	190.08 (28.86)
74 kg^^^	**182.10 (34.19)**^**‡**^	166.32 (32.19)	**119.40 (23.74)**^**‡**^	107.37 (21.99)	**217.88 (34.30)**^**‡**^	200.20 (31.23)
77 kg^#^	180.25 (39.58)	169.54 (38.38)	117.77 (24.53)*	110.07 (23.88)	214.87 (38.75)*	201.71 (37.07)
83 kg^^^	**200.55 (34.08)**^**‡**^	174.41 (30.19)	**129.92 (21.13)**^**‡**^	113.93 (20.26)	**235.05 (34.73)**^**‡**^	208.84 (31.75)
85 kg^#^	191.02 (38.41)*	178.58 (30.02)	125.34 (28.49)	118.54 (20.79)	**224.36 (33.38)**^**†**^	210.50 (31.07)
93 kg^^^	**212.76 (33.23)**^**‡**^	189.26 (30.51)	**141.15 (22.41)**^**‡**^	124.09 (20.42)	**248.94 (32.45)**^**‡**^	223.40 (31.71)
94 kg^#^	**215.80 (34.14)**^**‡**^	190.76 (31.87)	**141.50 (22.75)**^**‡**^	124.49 (21.16)	**249.99 (32.78)**^**‡**^	223.04 (32.22)
105 kg^#^^	**219.27 (34.86)**^**‡**^	199.00 (35.06)	**147.97 (26.14)**^**‡**^	132.37 (24.68)	**255.16 (34.47)**^**‡**^	231.37 (34.65)
120 kg^#^^	**227.71 (41.68)**^**‡**^	201.45 (39.52)	**148.97 (27.01)**^**‡**^	133.85 (26.62)	**256.48 (37.60)**^**‡**^	233.19 (34.60)
120 kg+^#^^	251.24 (68.63)	238.70 (60.57)	161.95 (37.55)	160.43 (37.77)	263.97 (48.92)	256.37 (46.67)

Data are mean (standard deviation). * P < 0.05, ^†^P < 0.01, ^‡^P < 0.001 when compared to lost. ^**#**^ World Powerlifting (WP), ^**^**^ International Powerlifting Federation (IPF)

**Fig. 1:**
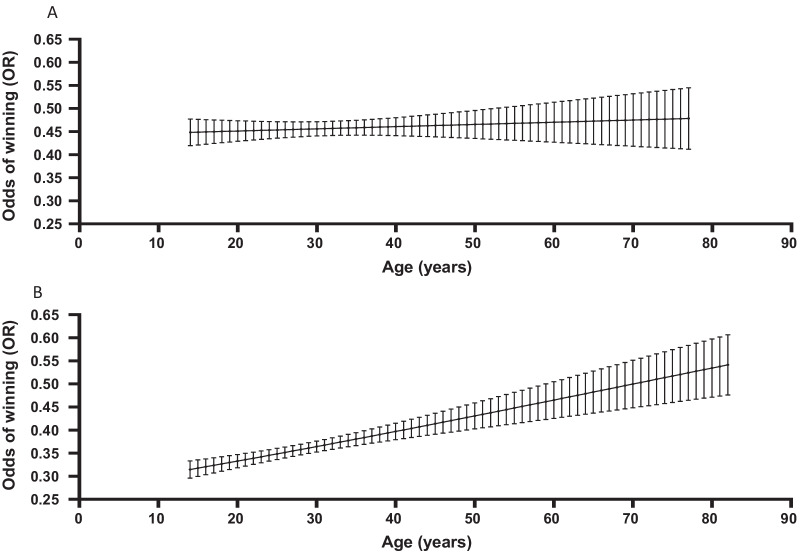
Shows data from International Powerlifting Federation and World Powerlifting and results for: **a** probability of winning by entrant age (years) for female competitors, and **b** probability of winning by entrant age (years) for male competitors

**Fig. 2:**
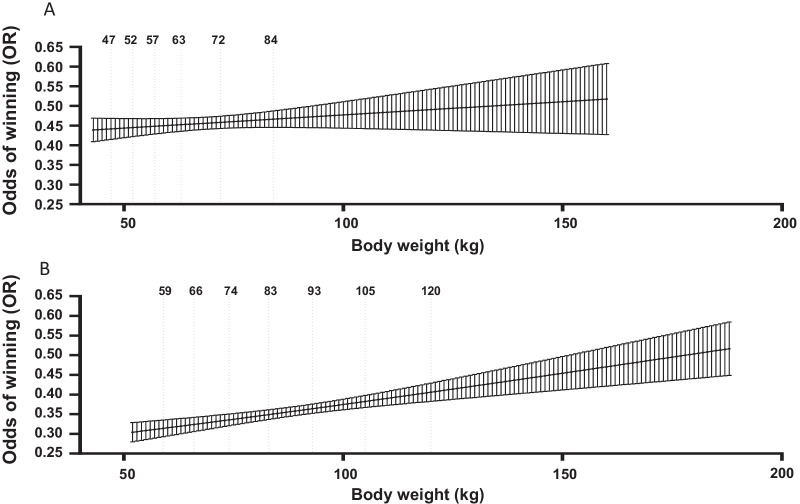
**a** Probability of winning by body weight (kg) for all female competitors (International Powerlifting Federation (IPF) female weight classes shown). **b** Probability of winning by body weight (kg) for all male competitors (International Powerlifting Federation (IPF) male weight classes shown)

For all competitors (i.e. females and males combined across all weight classes), the odds of winning were 1.1% greater (OR [95% CI] 1.011 [1.008, 1.015], P < 0.001; for each additional year of age. Moreover, the odds of winning were 1.4% greater (OR [95% CI] 1.014 [1.009, 1.019], P < 0.001) per additional year of age for males, but not females (P = 0.509; Fig. 1). When compared to males, the odds of winning for females were 50% greater (OR [95% CI] 1.500 [1.384, 1.625]; P < 0.001). When comparing male athletes across all weight classes, the odds of winning were 0.7% greater (OR [95% CI] 1.007 [1.004, 1.009], P < 0.001; (Fig. 2) per kilogram of additional body weight, but this effect was not observed for females (P = 0.180), or across the total sample (P = 0.403) (Table 3).

**Table 3:** Bodyweight comparisons of competitors included in analyses

Weight class	n	Body weight (kg)					d (95% CI)	P-value
		All: Minimum	All: Mean (SD)	All: Maximum	Won: Mean (SD)	Lost: Mean (SD)		
All	10,599	43.30	81.90 (19.87)	195.00	81.7 (22.6)	82.0 (17.8)	− 0.02 (− 0.06, 0.02)	0.403
Female	4032	43.30	69.52 (15.69)	160.00	69.88 (17.56)	69.22 (13.93)	0.04 (− 0.02, 0.10)	0.180
47 kg^#^	86	43.30	46.25 (0.74)	47.00	46.23 (0.77)	46.34 (0.67)	− 0.14 (− 0.64, 0.35)	0.565
48 kg^^^	24	44.00	47.29 (0.97)	48.00	47.25 (0.93)	47.37 (1.11)	− 0.12 (− 0.97, 0.73)	0.784
52 kg^#^	252	47.15	50.82 (1.07)	52.00	50.75 (1.16)	50.93 (0.93)	− 0.17 (− 0.42, 0.08)	0.183
53 kg^^^	64	48.75	51.62 (1.09)	52.95	51.48 (1.12)	51.85 (1.02)	− 0.34 (− 0.85, 0.17)	0.188
57 kg^#^	410	52.02	55.79 (1.07)	57.00	55.86 (1.05)	55.70 (1.09)	0.15 (− 0.05, 0.34)	0.133
58 kg^^^	133	53.20	56.73 (1.14)	58.00	56.77 (1.12)	56.71 (1.16)	0.06 (− 0.29, 0.40)	0.744
63 kg^#^	651	57.05	61.43 (1.33)	63.00	61.56 (1.27)	61.34 (1.37)	0.17 (0.01, 0.32)	0.040
64 kg^^^	213	58.35	62.54 (1.30)	64.00	62.66 (1.28)	62.48 (1.32)	0.14 (− 0.15, 0.42)	0.352
72 kg^#^^	1019	63.15	69.45 (2.09)	72.00	69.43 (2.14)	69.46 (2.07)	− 0.02 (− 0.15, 0.11)	0.800
84 kg^#^^	659	72.10	79.34 (3.23)	84.00	79.47 (3.31)	79.23 (3.16)	0.07 (− 0.08, 0.23)	0.346
84 kg + ^#^	395	84.20	100.22 (14.58)	160.00	100.68 (15.22)	99.64 (13.75)	0.07 (− 0.13, 0.27)	0.479
100 kg^^^	79	84.40	93.79 (4.40)	99.93	94.36 (4.41)	93.13 (4.37)	0.28 (− 0.17, 0.72)	0.219
100 kg + ^^^	47	100.45	112.96 (8.49)	137.55	112.65 (8.61)	113.61 (8.47)	− 0.11 (− 0.72, 0.50)	0.723
Male	6567	52.05	89.50 (18.30)	188.10	90.93 (21.83)	88.70 (15.91)	0.12 (0.07, 0.17)	< 0.001
59 kg^#^	142	52.05	57.74 (1.41)	59.00	57.76 (1.29)	57.65 (1.72)	0.08 (− 0.30, 0.46)	0.686
62 kg^^^	24	54.15	59.88 (1.90)	62.00	59.79 (1.94)	60.35 (1.87)	− 0.29 (− 1.37, 0.79)	0.598
66 kg^#^	296	59.55	64.89 (1.23)	66.00	64.98 (1.13)	64.76 (1.34)	0.18 (− 0.05, 0.41)	0.123
69 kg^^^	96	62.25	67.27 (1.54)	69.00	67.69 (1.15)	66.85 (1.76)	0.57 (0.16, 0.98)	0.006
74 kg^#^	853	66.05	72.42 (1.62)	74.00	72.60 (1.58)	72.33 (1.63)	0.17 (0.03, 0.31)	0.020
77 kg^^^	193	69.25	74.98 (1.72)	77.00	75.24 (1.72)	74.84 (1.71)	0.23 (− 0.07, 0.53)	0.131
83 kg^#^	1223	74.10	80.93 (1.94)	83.00	81.26 (1.68)	80.80 (2.01)	0.24 (0.11, 0.37)	< 0.001
85 kg^^^	205	77.55	82.75 (1.76)	85.00	83.01 (1.53)	82.61 (1.86)	0.22 (− 0.06, 0.51)	0.128
93 kg^#^	1328	83.08	90.06 (2.39)	93.00	90.31 (2.33)	89.98 (2.40)	0.14 (0.01, 0.26)	0.028
94 kg^^^	241	85.32	91.28 (2.16)	94.00	91.59 (2.04)	91.15 (2.20)	0.21 (− 0.07, 0.48)	0.145
105 kg^#^^	1042	93.10	100.78 (3.21)	105.00	101.13 (3.13)	100.58 (3.25)	0.17 (0.04, 0.30)	0.008
120 kg^#^^	590	105.10	114.07 (4.12)	120.00	114.08 (4.27)	114.06 (3.98)	0.00 (− 0.16, 0.17)	0.955
120 kg+^#^^	334	120.20	138.77 (15.24)	188.10	139.38 (16.30)	137.96 (13.77)	0.09 (− 0.12, 0.31)	0.400

SD, standard deviation; d, Cohen’s d effect size

^#^International Powerlifting Federation weight class, ^^^World Powerlifting weight class

Relative and absolute first SQ weight (regardless of success) for each weight division (IPF and WP classifications) is shown in Table 4. Irrespective of weight class, absolute first SQ attempt weight of females who won was 10.5 kg greater (P < 0.001, *d* = 0.44 [0.38, 0.50]) than those who lost, and each additional kilogram was associated to a 1.9% (OR [95% CI] 1.019 [1.016, 1.021], P < 0.001) increased odds of winning. In males who won, compared to those who lost, first SQ attempt weight was 16.8 kg greater (P < 0.001, *d* = 0.41 [0.36, 0.46]) and each additional kilogram corresponded to a 1.0% (OR [95% CI] 1.010 [1.009, 1.012], P < 0.001) greater odds of winning. Relative SQ attempt weight differed between winners and non-winners in eight out of thirteen weight classes for females and six out of thirteen weight classes for males. In the total sample, 67.25% of athletes who won achieved their maximum successful SQ at attempt three compared to 63.39% of non-winners (OR [95% CI; 1.19 [1.09, 1.29] p < 0.001) resulted in a significantly improved OR for winning an event. For female winners compared to non-winners (67.95% vs 62.76% (1.26 [1.10, 1.43] p < 0.01) and for male winners compared to non-winners (66.71% vs 63.72% (1.14 [1.03, 1.27] p < 0.05) these success rates also resulted in significantly improved OR for winning an event.

**Table 4:** Average absolute and relative first lift weight between those who won and lost

Variable		Squat				Bench Press				Deadlift			
	n	Absolute		Relative		Absolute		Relative		Absolute		Relative	
		Win	Lose	Win	Lose	Win	Lose	Win	Lose	Win	Lose	Win	Lose
Female													
47 kg^#^	86	80.4 (18.8)	71.5 (15.2)	1.46 (1.03)	1.33 (0.87)	46.9 (10.9)	44.3 (11.8)	0.78 (0.69)	0.87 (0.48)	103.7 (15.9)*	92.4 (20.0)	2.10 (0.86)*	1.57 (1.34)
48 kg^^^	24	91.4 (22.6)	88.9 (13.3)	1.34 (1.51)	1.45 (1.30)	54.7 (11.8)	52.0 (11.2)	1.02 (0.61)	1.10 (0.23)	114.8 (22.8)	111.8 (15.7)	2.05 (1.42)	2.36 (0.34)
52 kg^#^	252	**92.3 (19.8)**^**†**^	84.6 (16.7)	**1.58 (0.97)**^**‡**^	1.03 (1.35)	**52.6 (11.2)**^**†**^	48.4 (9.4)	**0.94 (0.50)**^**†**^	0.73 (0.64)	**113.2 (21.3)**^**†**^	105.9 (19.2)	2.11 (0.84)*	1.79 (1.12)
53 kg^^^	64	90.1 (16.7)	88.3 (18.0)	1.64 (0.69)	1.21 (1.26)	50.1 (10.5)	48.3 (9.6)	0.87 (0.48)	0.82 (0.49)	112.0 (18.0)	114.0 (20.8)	2.18 (0.35)*	1.66 (1.52)
57 kg^#^	410	**97.3 (20.6)**^**‡**^	88.4 (20.4)	**1.48 (0.99)**^**†**^	1.15 (1.16)	**56.7 (11.8)**^**‡**^	50.9 (12.1)	**0.89 (0.52)**^**†**^	0.76 (0.55)	**120.5 (21.3)**^**‡**^	110.8 (22.4)	2.00 (0.89)*	1.79 (0.94)
58 kg^^^	133	**100.0 (18.7)**^**†**^	91.6 (14.3)	1.32 (1.22)	1.28 (1.02)	56.5 (12.9)*	51.7 (9.6)	**0.99 (0.22)**^**†**^	0.77 (0.52)	**123.4 (20.2)**^**†**^	113.9 (17.6)	2.02 (0.88)	1.78 (0.98)
63 kg^#^	651	**103.8 (21.6)**^**‡**^	93.5 (19.6)	**1.47 (0.90)**^**†**^	1.25 (0.93)	**58.9 (12.6)**^**‡**^	52.9 (11.3)	0.85 (0.49)*	0.75 (0.46)	**126.9 (22.8)**^**‡**^	114.7 (21.0)	1.86 (0.96)	1.77 (0.70)
64 kg^^^	213	**110.0 (24.6)**^**‡**^	98.0 (21.3)	1.65 (0.71)*	1.37 (0.83)	**60.6 (12.8)**^**‡**^	53.6 (11.7)	0.90 (0.41)*	0.76 (0.43)	**132.0 (25.6)**^**‡**^	117.1 (23.8)	**2.11 (0.40)**^**‡**^	1.84 (0.50)
72 kg^#^^	1019	**112.6 (22.2)**^**‡**^	98.5 (21.1)	**1.39 (0.89)**^**‡**^	1.13 (0.90)	**61.6 (12.2)**^**‡**^	54.7 (11.8)	**0.80 (0.42)**^**‡**^	0.69 (0.41)	**134.6 (23.3)**^**‡**^	119.1 (21.8)	**1.87 (0.62)**^**‡**^	1.63 (0.60)
84 kg^#^^	659	**114.7 (24.1)**^**‡**^	101.4 (24.1)	**1.18 (0.87)**^**†**^	0.99 (0.86)	**63.8 (14.6)**^**‡**^	57.6 (13.5)	**0.71 (0.41)**^**†**^	0.63 (0.40)	**134.4 (23.1)**^**‡**^	121.5 (22.5)	**1.64 (0.49)**^**‡**^	1.45 (0.57)
84 kg + ^#^	395	**120.8 (32.4)**^**‡**^	107.5 (26.9)	0.89 (0.87)*	0.65 (0.91)	**66.4 (15.1)**^**‡**^	60.9 (13.5)	0.61 (0.31)*	0.53 (0.34)	**138.3 (27.6)**^**‡**^	125.4 (22.6)	1.29 (0.57)	1.22 (0.42)
100 kg^^^	79	121.2 (26.5)*	106.4 (25.4)	1.29 (0.28)*	0.90 (0.76)	**68.9 (15.3)**^**†**^	59.7 (13.9)	**0.73 (0.16)**^**†**^	0.51 (0.41)	137.6 (22.8)	127.9 (28.1)	1.46 (0.23)	1.37 (0.27)
100 kg+^^^	47	125.3 (34.3)	113.4 (16.0)	0.83 (0.82)	0.46 (0.94)	68.0 (14.6)	64.1 (6.3)	0.61 (0.13)	0.57 (0.07)	131.5 (18.9)	124.3 (13.8)	1.17 (0.18)	1.10 (0.13)
Male													
59 kg^#^	142	127.4 (32.6)	135.8 (42.3)	**1.93 (1.21)**^**†**^	0.93 (2.32)	84.7 (18.9)	82.7 (15.2)	1.21 (0.89)	1.17 (0.87)	156.1 (30.6)	154.7 (36.3)	2.31 (1.50)	2.10 (1.78)
62 kg^^^	24	100.7 (37.9)*	150.6 (62.4)	1.48 (1.00)*	− 0.59 (3.00)	72.6 (26.0)	94.1 (26.0)	0.88 (0.95)	1.55 (0.40)	138.3 (42.0)	161.3 (26.3)	1.81 (1.61)	1.37 (2.68)
66 kg^#^	296	143.1 (31.0)	139.1 (28.2)	1.78 (1.38)	1.83 (1.19)	93.3 (19.7)	90.2 (18.8)	1.16 (0.89)	1.13 (0.86)	175.6 (33.9)	169.5 (29.9)	2.47 (1.20)	2.27 (1.38)
69 kg^^^	96	141.3 (35.3)	144.5 (30.9)	1.95 (0.91)	1.57 (1.56)	89.7 (17.3)	92.1 (19.0)	1.23 (0.56)	1.12 (0.85)	171.5 (36.3)	172.6 (28.7)	2.20 (1.37)	2.23 (1.37)
74 kg^#^	853	**169.0 (33.5)**^**‡**^	153.9 (32.2)	1.80 (1.55)	1.75 (1.28)	**112.2 (23.0)**^**‡**^	100.5 (21.3)	1.27 (0.93)*	1.11 (0.88)	**200.6 (33.7)**^**‡**^	185.0 (32.1)	2.41 (1.42)*	2.15 (1.45)
77 kg^^^	193	166.5 (36.5)*	155.0 (35.6)	1.69 (1.51)	1.75 (1.20)	109.2 (23.8)*	101.5 (23.0)	1.20 (0.88)	1.13 (0.81)	196.5 (37.2)	185.7 (37.3)	2.34 (1.26)	2.19 (1.26)
83 kg^#^	1223	**185.7 (32.5)**^**‡**^	161.1 (29.4)	**1.92 (1.30)**^**‡**^	1.59 (1.25)	**121.9 (21.0)**^**‡**^	106.5 (20.1)	**1.37 (0.66)**^**‡**^	1.12 (0.74)	**217.8 (33.4)**^**‡**^	192.4 (32.0)	**2.44 (1.17)**^**‡**^	2.14 (1.11)
85 kg^^^	205	**175.6 (37.4)**^**†**^	162.7 (29.9)	1.48 (1.59)	1.69 (1.07)	116.3 (26.9)*	109.5 (20.5)	1.13 (0.89)	1.23 (0.54)	**206.5 (32.1)**^**†**^	191.8 (30.2)	2.25 (1.13)	2.16 (0.92)
93 kg^#^	1328	**197.1 (31.9)**^**‡**^	174.8 (30.5)	**1.86 (1.19)**^**‡**^	1.56 (1.21)	**131.5 (21.5)**^**‡**^	115.9 (20.3)	**1.26 (0.76)**^**‡**^	1.05 (0.77)	**230.7 (33.0)**^**‡**^	206.2 (32.4)	**2.43 (0.85)**^**‡**^	2.11 (0.97)
94 kg^^^	241	**197.8 (32.8)**^**‡**^	175.2 (30.8)	**1.98 (0.94)**^**†**^	1.48 (1.27)	**131.5 (22.5)**^**‡**^	115.5 (21.1)	1.36 (0.51)*	1.18 (0.51)	**231.3 (33.1)**^**‡**^	203.8 (32.6)	2.33 (1.05)	2.20 (0.55)
105 kg^#^^	1042	**201.6 (33.6)**^**‡**^	182.9 (34.2)	1.66 (1.15)	1.54 (1.02)	**138.0 (25.7)**^**‡**^	123.7 (24.2)	**1.19 (0.70)**^**†**^	1.06 (0.66)	**234.7 (33.8)**^**‡**^	213.2 (34.9)	**2.17 (0.89)**^**‡**^	1.94 (0.92)
120 kg^#^^	590	**209.3 (41.0)**^**‡**^	184.4 (40.6)	**1.49 (1.12)**^**†**^	1.24 (1.09)	**139.4 (27.3)**^**‡**^	124.6 (26.1)	**1.06 (0.65)**^**†**^	0.90 (0.66)	**235.2 (37.4)**^**‡**^	214.5 (35.0)	**2.00 (0.59)**^**‡**^	1.70 (0.87)
120 kg+^#^^	334	230.2 (65.5)	220.8 (60.1)	1.49 (0.81)	1.30 (1.03)	150.4 (36.2)	149.4 (36.6)	0.95 (0.56)	0.87 (0.70)	242.4 (49.5)	237.4 (48.7)	1.65 (0.69)	1.49 (0.95)

Data are mean (standard deviation)

Relative weight attempt = Absolute weight (kg)/body mass (kg)

^*^P < 0.05; ^†^P < 0.01; ^‡^P < 0.001 when compared to lose

^#^International Powerlifting Federation weight class, ^^^World Powerlifting weight class

For each weight division (IPF and WP classifications), relative and absolute first BP weight (regardless of success) is shown in Table 4. Relative BP attempt weight differed between winners and non-winners in nine out of thirteen weight classes for females and six out of thirteen weight classes for males. For the BP, irrespective of weight division opening attempt weights for females who won were 5.6 kg greater (P < 0.001, *d* = 0.43 [0.37, 0.49]) and reflected a 3.3% (OR [95% CI] 1.033 [1.028, 1.038], P < 0.001) greater odds of winning for each additional kilogram. For males, absolute first BP attempt weight of those who won was 11.3 kg greater (P < 0.001, *d* = 0.42 [0.36, 0.47]) than those who lost and each additional kilogram was associated with a 1.5% (OR [95% CI] 1.015 [1.013, 1.017], P < 0.001) increased odds of winning. In the total sample, 49.46% of athletes who won achieved their maximum successful BP at attempt three compared to 46.77% of non-winners (OR [95% CI; 1.11 [1.03, 1.20] p < 0.01). For female winners compared to non-winners (47.69% vs 44.91% (1.12 [0.99, 1.27] p > 0.05) the OR for success did not differ significantly. For male winners compared to non-winners (50.85% vs 47.73% (1.13 [1.02, 1.25] p < 0.05) the success rate of the BP significantly improved the OR for competitive success.

In Table 4, relative and absolute first DL weight (regardless of success) is shown for each weight class (IPF and WP classifications). Relative DL attempt weight differed between winners and non-winners in seven out of thirteen weight classes for females and five out of thirteen weight classes for males. In females who won, compared to those who lost, first DL attempt weight was 10.9 kg greater (P < 0.001, *d* = 0.47 [0.40, 0.53]) and each additional kilogram corresponded to a 2.0% (OR [95% CI] 1.020 [1.017, 1.023], P < 0.001) greater odds of winning. For males, absolute first DL attempt weight of those who won was 16.5 kg greater (P < 0.001, *d* = 0.42 [0.37, 0.47]) than those who lost and each additional kilogram was associated with a 1.1% (OR [95% CI] 1.011 [1.010, 1.012], P < 0.001) increased odds of winning.

In the total sample, 62.61% of athletes who won achieved their maximum successful DL at attempt three compared to 61.42% of non-winners (OR [95% CI; 1.05 [0.97, 1.14] p > 0.05). For female winners compared to non-winners (67.84% vs 67.64% (1.01 [0.88, 1.15] p > 0.05) the OR for success did not differ significantly. For male winners compared to non-winners (58.52% vs 58.18% (1.01 [0.92, 1.12] p > 0.05) the success rate of the BP did not significantly improve the OR for competitive success.

## Discussion

The aim of this investigation was to report differences in the successful lift weights of winners and non-winners, and explore factors directly related to competition that may influence or contribute to success for PL athletes. Specifically, we reported the average winning weight for each lift in each weight class. We also determined the OR of winning a PL competition based on univariate analysis of age, sex, bodyweight, length of time competing and opening attempt weight selection relative to bodyweight for the SQ, BP and DL. The results suggest that competitors who are heavier (males only), older, have larger first lift attempts and have competed for a longer period have an increased likelihood winning compared to other PL athletes. This information is intended to help expand the evidence base in the sport of PL and assist coaches and athletes in competition tactics and strategy to increase the likelihood of successful performance.

Successful completion of the first attempt in any lift has previously been shown to be greater for PL athletes who placed in the top three of their weight class and discipline compared to those who did not [15]. This may lend to the thinking that athletes should choose a conservative weight that they are confident in making for the first attempt. However, our results suggest that irrespective of attempt success, the weight selection of the first attempt is an important variable in the overall competition result, and likely serves as an indicator of overall athlete strength. Previous work by Howells et al. [12] has shown that regardless of weight class, and attempt success, the absolute first SQ attempt weight of those who won compared to those who lost was 16.8 kg and 10.5 kg greater for males and females respectively and resulted in a greater chance of winning. Additionally, the current data also show that the BP (Males: 11.3 kg, Females 5.6 kg) and DL (Males: 16.5 kg Females: 10.9 kg greater) follow a similar trend, suggesting that greater opening attempt weights may serve as a predictor of competitive success. Travis et al. [11] reported that attempt weights for elite powerlifters who successfully completed their opening SQ attempt were approximately 91% of their one repetition maximum. Similarly, the increase between attempts or final attempt weights is of consideration. Our data show that athletes and coaches typically select final attempt weights for the SQ and DL which are achievable by most athletes (attempt success > 60% for both winners and non-winners). However, less than 50% of athletes achieve a successful lift at their third attempt for the BP. This may signal that attempting too great an increase between attempt two and three minimizes the opportunity to increase competition total. A more radical approach to competition would be to adopt an “all or nothing” strategy. For example, if the intention is to win (rather than focus on setting a personal best), athletes and coaches may utilise a more aggressive competition approach based on the average winning attempt weights for each lift in a respective class (see Table 4). Theoretically, this would see all attempts made at, or close to this value. However, such a strategy may increase the odds of an unsuccessful first and subsequent attempts. Second, maximum competition scores are only known post completion and may differ between individual competitions based on the athletes competing. For these reasons, coaches may consider a moderated strategy which targets opening attempts above the 91% of one repetition maximum typically used, in favour of attempts weights typically chosen at attempt two instead (i.e. ~ 95–97% of one repetition maximum). We acknowledge that this suggestion differs from current coaching strategies, thus we recommend that the adoption of such an approach (based on the current results) should be trialled during training and/or recreational competitions prior to possible implementation at higher level tournaments.

These results suggest that stronger, winning athletes are also more experienced. Specifically, those who won had participated over a greater time period (~ 100 days longer) and in more events on average than their counterparts who did not win (3.4 vs 2.8 competitions), and subsequently, may be more confident and better prepared (mentally and physically) to select greater opening attempt weights. We suggest that this added exposure to competition may assist to out strategize and gain initial advantage, both mental and score-based, over less experienced competitors. For example, gaining early psychological advantage may be of particular importance as previous research in college PL athletes showed a significant negative relationship between competition anxiety and competition total [18]. Additionally, emotional stress (anxiety and low mood) and fatigue are reported as the most influential competition factors among Russian powerlifters of both sexes [19]. Howells et al. [12] showed that the increase in weight between SQ attempts of non-winners trended parallel to those of winners (i.e. non-winners did not reduce the margin between themselves and winners which was established at the first attempt). Similarly, Coker et al. [10] highlight the effects of increasing the number of successful lifts in achieving competitive success with a greater number of successful attempts associated with better competitive outcomes. Thus, it may prove useful for less strong athletes to preference a more substantial opening attempt to minimise any early lead developed by stronger athletes using more conservative opening attempts. Additionally, coaches should also consider that the SQ and DL present the greatest opportunities to expand the margin between winners and non-winners in terms of kilograms lifted [10]. Further, small differences in attempt selection may drastically influence competition outcomes. Thus, coaches may choose to preference the development of the SQ and DL to further enhance the likelihood of success for their athletes. It is important to note that there are some weight classes where there is no statistical difference between maximum, successful attempt weights of winners compared to non-winners for any or all of the SQ, BP or DL. The absence of statistical significance here must not be interpreted as no meaningful difference in terms of competition. Given that competitions are won based on the largest absolute total (kg) lifted by an athlete in each weight class, coaches may identify these classes as highly competitive and may preference favourable body weight changes to transition to less competitive class and to positively improve performance outcomes within such classes.

The results of this investigation also suggest that each additional year of age of an athlete increases their likelihood of winning an event. This finding may, at least in part, be explained by the reduced number of athletes competing beyond the “open” age category. As athletes progress to “masters” competition (e.g. aged 40 years or over), there are fewer competitors (11% and 19% of total male and female competitors, respectively) and therefore, the likelihood of winning is increased irrespective of actual strength capability. But this does not diminish the role of training age. In support of training age influencing success, those who won competitions had typically competed for a longer period than those who lost (427 vs 318 days) with similar observations within each sex (refer to Table 1). Thus, athletes who begin competing either at a younger age, or who sustain participation in PL for a longer period may have a distinct advantage. Moreover, it can be postulated that they are likely to have developed greater skill (e.g. movement task specific and tactical ability) and neuromuscular adaptations prior to reaching peak muscle mass, or those which can be maintained with increased age to assist winning competitions in later years [8, 20–22].

The odds of winning for females was 1.5 times greater when compared to males. Importantly, there were an equal number of competitions and therefore, a similar amount of opportunity to win in much of the current data set. Thus, like “masters” athletes, this result is likely to be largely explained by the reduced number of female competitors compared to males. For example, there were ~ 40% less female (n = 4032) competitors compared to males (n = 6569), suggesting an increased odd of winning for females just by partaking in competition. Additionally, the recent transition of Powerlifting Australia from the IPF [1] to WP [23] increased the total number of competitive weight classes for females from seven to eight. This transition created an additional competitive class for females and spread the number of athletes across a greater number of classes, reducing competition within a given class even further. When combined, these factors lead to the logical conclusion that due to equal number of opportunities for success, female athletes have a greater likelihood of winning simply because they have fewer competitors.

Our findings also show that increasing body weight results in an increased odd of winning for males but not females or across the total sample. Previous works have shown that PL athletes with greater body weight possess greater absolute strength [24, 25]. In the heaviest weight categories (i.e. those without an upper weight limit), it is apparent that increasing body weight will generally increase the “total” weight lifted [13]. Similarly, being closer to the maximum allowable weight within a capped weight classes is often associated with greater strength [10]. However, the body weight of those who won compared to those who lost did not typically differ within weight classes. This suggests that athletes should consider optimising body composition (fat mass to lean mass ratio). The lack of difference in bodyweight between winners and non-winners within weight classes in our results may be explained by known reductions in lean- to fat-mass ratio that can occur as weight increases [9, 26]. In particular, fat free mass is positively correlated with PL performance [25], and may be the greatest anthropometric determinant of maximal strength [24, 25, 27, 28]. Therefore, body composition may have a greater influence on results than total body weight within weight classes, however, we were unable to directly test this in the current investigation. As there are fewer athletes who compete in the lightest and heaviest weight classes, it is possible that the relationship between odds of winning and body weight is non-linear. As such, athletes who compete at the weight class “tails” may have greater odds of winning than those in weight classes with greater participant numbers, but simple logistic regression may not fully capture this relationship.

Whilst rigorous, the present study is subject to some limitations. First, analysis of the effects of bodyweight on competition outcomes is unable to account for differences in the body composition (i.e., fat mass and fat free mass) of competitors. Second, the analyses conducted in this investigation included some participants who competed more than once, thus violating the assumption of independence. However, the results of this investigation are designed to be inferential rather than predictive. These data should serve to inform future athletes of the trends and inferences as they relate to their own competition. Future analyses should consider a multivariate analysis that explores the potential relationships between age, time competing, and other factors in elite and sub-elite competitors where winning is the priority. Third, only data from Australian IPF sanctioned PL competitions were analysed in this investigation. The trends and inferences from these data may differ in IPF and alternate PL federations competitions globally.

Collectively, the results presented in this article support our original hypotheses. For example, in many weight classes opening attempt weights across the lifts were greater for eventual winners than non-winners. Additionally, we also confirmed that competitors who are heavier (males only), older, have larger first lift attempts and have competed for a longer period have an increased likelihood winning compared to other PL athletes. Whilst the findings appear somewhat intuitive, this investigation has provided empirical evidence to support the anecdotal train of thought of coaches and athletes, added to the limited evidence base in the sport, and generally complemented the few published works regarding PL competition performance. Importantly, the present study has considered data from various competition levels over a substantial period making these findings comprehensive and unique in that they are applicable outside of just elite competition(s). Indeed, the purpose of this study was to help coaches and athletes develop better strategies and tactical decisions to increase the odds of competitive success. We realise that elite PL athletes only make up a small portion of the entire competitor pool, and that those trying to progress through the ranks will likely benefit immensely from such information as well. Thus, the information provided here serves to inform coaches working with athletes of all levels.

## Practical applications

- The selection of opening attempt weights for the SQ, BP and DL have significant implications for improving the odds of competitive success in PL.
- Coaches may target athlete training to achieve average winning scores in one or more disciplines.
- Coaches should consider increasing athletes’ exposure to competition or competition-like simulation to improve their ability to perform successful lifts at the heaviest possible loads under competition conditions.
- Athletes and coaches might consider implementing opening attempts which are at or near their perceived maximum within training and simulated competition prior to use in formal events.

## Data Availability

The datasets generated and/or analysed during the current study are available in the Openpowerlifting.org repository, https://openpowerlifting.gitlab.io/opl-csv/

## Abbreviations

BP: Bench press
CI: Confidence interval
d: Cohen’s d effect size
DL: Deadlift
IPF: International Powerlifting Federation
OR: Odds ratio
PL: Powerlifting
SQ: Squat
WP: World powerlifting

## References

[1] International Powerlifting Federation. Technical rules book, vol. 2019. http://www.powerlifting-ipf.com/fileadmin/ipf/data/rules/technical-rules/english/IPF_Technical_Rules_Book_2016__1_.pdf: IPF; 2016.

[2] Schoenfeld BJ, Grgic J, Ogborn D, Krieger JW (2017). Strength and hypertrophy adaptations between low- vs. high-load resistance training: a systematic review and meta-analysis. J Strength Cond Res.

[3] Colquhoun RJ, Gai CM, Walters J, Brannon AR, Kilpatrick MW, D’agostino DP (2017). Comparison of powerlifting performance in trained men using traditional and flexible daily undulating periodization. J Strength Cond Res.

[4] Ferland PM, Comtois AS (2019). Classic powerlifting performance: A systematic review. J Strength Cond Res.

[5] Abe T, Buckner SL, Mattocks KT, Jessee MB, Dankel SJ, Mouser JG, et al. Skeletal muscle mass and architecture of the world’s strongest raw powerlifter: a case study. Asian J Sports Med. 2018;9(2).

[6] Keogh JWLL, Hume PA, Pearson SN, Mellow PJ (2009). Can absolute and proportional anthropometric characteristics distinguish stronger and weaker powerlifters?. J Strength Cond Res.

[7] Ye X, Loenneke JP, Fahs CA, Rossow LM, Thiebaud RS, Kim D (2013). Relationship between lifting performance and skeletal muscle mass in elite powerlifters. J Sports Med Phys Fitness.

[8] Pearson J, Spathis JG, van den Hoek DJ, Owen PJ, Weakley J, Latella C (2020). Effect of competition frequency on strength performance of powerlifting athletes. J Strength Cond Res.

[9] Latella C, van den Hoek D, Teo W-P (2019). Differences in strength performance between novice and elite athletes: Evidence from powerlifters. J Strength Cond Res.

[10] Coker NA, Varanoske AN, Baker KM, Hahs-Vaughn DL, Wells AJ (2018). Predictors of competitive success of national-level powerlifters: a multilevel analysis. Int J Perform Anal Sport.

[11] Travis SK, Zourdos MC, Bazyler CD (2020). Weight selection attempts of elite classic powerlifters. Percept Mot Skills.

[12] Howells RJ, Spathis JG, Pearson J, Latella C, Garrett JM, Owen PJ (2022). Impacts of squat attempt weight selection and success on powerlifting performance. J Sports Med Phys Fitness..

[13] Latella C, van den Hoek D, Teo W-P (2018). Factors affecting powerlifting performance: an analysis of age- and weight-based determinants of relative strength. Int J Perform Anal Sport.

[14] Latella C, Owen PJ, Davies T (2022). Long-term adaptations in the squat, bench press and deadlift: assessing strength gain in powerlifting athletes. Med Sci Sports Exerc..

[15] Pritchard HJ, Morton RH (2015). Powerlifting: success and failure at the 2012 Oceania and 2013 classic world championships. J Aust Strength Cond.

[16] Cohen J (1992). A power primer. Psychol Bull.

[17] Drachman D (2012). Adjusting for multiple comparisons. J Clin Res Best Pract..

[18] Judge LW, Urbina LJ, Hoover DL, Craig BW, Judge LM, Leitzelar BM (2016). The impact of competitive trait anxiety on collegiate powerlifting performance. J Strength Cond Res.

[19] Ljdokova GM, Ismailova NI, Panfilov AN, Farhatovich KA (2015). Gender aspects of confounding factors in the preparation of powerlifters. Biosci Biotechnol Res ASIA.

[20] Falk B, Tenenbaum G (1996). The effectiveness of resistance training in children. Sport Med.

[21] Ozmun JC, Mikesky AE, Surburg PR (1994). Neuromuscular adaptations following prepubescent strength training. Med Sci Sport Exerc.

[22] Latella C, Teo W-P, Spathis J, van den Hoek D (2020). Long-term strength adaptation: a fifteen year analysis of powerlifting athletes. J Strength Cond Res.

[23] World Powerlifting. World Powerlifting rules of competition. https://worldpowerlifting.com/rules/rules-english/. World Powerlifting Ltd; 2020.

[24] Keogh JWL, Hume PA, Pearson SN, Mellow P (2007). Anthropometric dimensions of male powerlifters of varying body mass. J Sports Sci.

[25] Brechue WF, Abe T (2002). The role of FFM accumulation and skeletal muscle architecture in powerlifting performance. Eur J Appl Physiol.

[26] Kraemer WJ, Torine JC, Silvestre R, French DN, Ratamess NA, Spiering BA (2005). Body size and composition of National Football League players. J Strength Cond Res.

[27] Mayhew JL, Ball TE, Ward TE, Hart CL, Arnold MD (1991). Relationships of structural dimensions to bench press strength in college males. J Sports Med Phys Fitness.

[28] Mayhew JL, Piper FC, Ware JS (1993). Anthropometric correlates with strength performance among resistance trained athletes. J Sports Med Phys Fitness.

